# Transcriptional signatures of Itk-deficient CD3^+^, CD4^+ ^and CD8^+ ^T-cells

**DOI:** 10.1186/1471-2164-10-233

**Published:** 2009-05-18

**Authors:** K Emelie M Blomberg, Nicole Boucheron, Jessica M Lindvall, Liang Yu, Julia Raberger, Anna Berglöf, Wilfried Ellmeier, CI Edvard Smith

**Affiliations:** 1 Department of Laboratory Medicine, Clinical Research Center, Karolinska Institutet, Karolinska University Hospital Huddinge, SE-141 86 Huddinge, Sweden; 2 Division of Immunobiology, Institute of Immunology, Center for Physiology, Pathophysiology and Immunology, Medical University of Vienna, A-1090 Vienna, Austria; 3 Department for Informatics, Center for Bioinformatics, Post box 1080 Blindern NO-0316, Oslo, Norway

## Abstract

**Background:**

The Tec-family kinase Itk plays an important role during T-cell activation and function, and controls also conventional versus innate-like T-cell development. We have characterized the transcriptome of Itk-deficient CD3^+ ^T-cells, including CD4^+ ^and CD8^+ ^subsets, using Affymetrix microarrays.

**Results:**

The largest difference between Itk^-/- ^and Wt CD3^+ ^T-cells was found in unstimulated cells, e.g. for killer cell lectin-like receptors. Compared to anti-CD3-stimulation, anti-CD3/CD28 significantly decreased the number of transcripts suggesting that the CD28 co-stimulatory pathway is mainly independent of Itk. The signatures of CD4^+ ^and CD8^+ ^T-cell subsets identified a greater differential expression than in total CD3^+ ^cells. Cyclosporin A (CsA)-treatment had a stronger effect on transcriptional regulation than Itk-deficiency, suggesting that only a fraction of TCR-mediated calcineurin/NFAT-activation is dependent on Itk. Bioinformatic analysis of NFAT-sites of the group of transcripts similarly regulated by Itk-deficiency and CsA-treatment, followed by chromatin-immunoprecipitation, revealed NFATc1-binding to the *Bub1*, *IL7R, Ctla2a*, *Ctla2b*, and *Schlafen1 *genes. Finally, to identify transcripts that are regulated by Tec-family kinases in general, we compared the expression profile of Itk-deficient T-cells with that of Btk-deficient B-cells and a common set of transcripts was found.

**Conclusion:**

Taken together, our study provides a general overview about the global transcriptional changes in the absence of Itk.

## Background

The Tec family of non-receptor protein-tyrosine kinases consists of five members (Bmx, Btk, Itk, Rlk/Txk and Tec); T-cells express Itk, Rlk and Tec, and T-cell receptor (TCR) stimulation leads to the activation of Tec family kinases [1,2]. A large number of biochemical studies and the generation of mice that are single- or double-deficient for Itk, Tec or Rlk have identified important roles, in particular for Itk, during T-cell development and activation, and in Th2 effector differentiation. Itk^-/- ^mice show impaired positive selection of CD4^+ ^T-cells and it was suggested that Itk modulates signaling thresholds during T-cell development [3-5]. TCR signaling in naïve T-cells, and therefore activation and proliferation, is impaired in the absence of Itk, and *Itk*^-/- ^T-cells show defective Th2 polarization [6]. Further, Itk regulates the actin cytoskeleton and is therefore necessary for proper synapse formation and for efficient T-cell activation [7,8]. More recent data indicate that Itk is involved in signaling pathways that regulate conventional versus innate-like T-cell development. The majority of CD8^+ ^T-cells from Itk^-/- ^as well as from Itk^-/-^Rlk^-/- ^mice show a more "innate-like" T-cell phenotype, sharing characteristics with conventional memory T-cells, i.e. CD44^hi^, CD62L^- ^and CD122^hi ^[9-11]. These cells depend on IL-15, express TCRs specific for non-classical MHC class Ib molecules, and exhibit direct effector functions such as rapid IFNγ production upon PMA/ionomycin stimulation [9-12]. A significant fraction of innate-like CD44^hi^CD62L^- ^T-cells has also been described for the CD4^+ ^T-cell lineage in Itk^-/- ^mice [13].

Biochemically, the defects in T-cell activation were linked to an impaired phospholipase C-γ (PLCγ) phosphorylation and activation [5]. PLCγ hydrolyzes phosphatidylinositol-4,5-biphosphate to produce inositol-1,4,5-triphosphate (IP_3_) and diacylglycerol (DAG). IP_3 _induces the release of intracellular calcium (Ca^2+^) thereby activating the serine/threonine phosphatase calcineurin. Itk activation results in high levels of IP_3_, which is required for Ca^2+ ^entry via store-operated channels leading to increased Ca^2+ ^in cells stimulated via the TCR [5]. Both Ca^2+ ^and calmodulin will bind and activate calcineurin, which in turn dephosphorylates serines in the regulatory domain of cytosolic NFAT. This induces a conformational change in NFAT exposing nuclear localization signals allowing its transport into the nucleus [14]. In Itk-deficient T-cells the Ca^2+^-levels are reduced resulting in impaired NFAT translocation [6]. Mice deficient in NFAT family members share phenotypes with Tec kinase family-deficient mice, as described by Lucas *et al*. [15]. The NFAT family was first described as binding to and controlling the interleukin 2 (IL-2) promoter and other lymphokine promoters in T-cells [14]. The family consists of five members; NFATc1–4 and NFAT5 [16,17]. Efficient inhibitors for the activation of NFAT proteins have been developed. Two of these, Cyclosporin A (CsA) and FK506, indirectly inhibit NFAT by blocking the enzymatic activity of calcineurin.

In order to further decipher the role of Itk we have investigated changes in gene expression of CD3^+ ^as well as CD4^+ ^and CD8^+ ^T-lymphocytes in normal and Itk-defective mice. The aim of the study was to (1) define the transcriptome in unstimulated cells, (2) elucidate the influence of anti-CD3 and anti-CD3/CD28-stimulation and (3) to dissect which part of the observed alterations in Itk-deficiency is dependent on the calcineurin/NFAT pathway.

## Methods

### Mice and generation of T-cells

CD3^+ ^as well as CD4^+ ^and CD8^+ ^T-cells from pooled suspensions of spleen and lymph nodes of Wt and Itk^-/- ^mice on C57BL/6 background were isolated by negative depletion; antibodies used are listed in Additional file 1. The cell suspensions were incubated with the antibodies in PBS supplemented with 2% FCS. Streptavidin beads (BD Pharmingen) were used for negative depletion according to manufacturer's instructions. The purity of the cells was assessed by flow cytometry and was routinely >90% CD3^+^, >96% CD4^+ ^and >90% CD8^+ ^T-cells. All animal experiments were approved by the Federal Ministry for Science and Research.

### T-cell stimulations and Cyclosporin A treatment

Unstimulated as well as stimulated T-cells were studied. Stimulations were performed in 48-well plates, coated with anti-CD3 (1 μg/ml) with or without anti-CD28 (3 μg/ml) in the presence or absence of CsA (1 μg/ml) for 24 hrs. For each stimulus, at least duplicate samples were used in all but one experiment. For the CD4^+ ^T-cells we collected triplicates from the Itk^-/- ^mice and duplicates from the Wt group. For the CD8^+ ^T-cells, we got duplicates from Itk^-/-^, while we obtained a single sample from Wt owing to the low cell yield for resting Wt CD8^+ ^T-cells. After anti-CD3-stimulation we got a single sample from the CD8^+ ^subset of both Wt and Itk^-/-^, while for the CD4^+ ^subsets we collected duplicates. To control if the number of differentially expressed probe-sets was truthful in the CD8^+ ^subset, and not due to the lack of replicates, we analyzed the CD4^+ ^in the same way as the CD8^+ ^T-cells. The results were consistent and we found the same number of differentially expressed transcripts when single CD4^+ ^samples were studied separately.

### RNA isolation and microarray processing

RNA isolations were done according to RNeasy Mini protocol (Qiagen, Valencia, CA, USA) and microarray processing as previously described [18]. The Affymetrix MOE430 2.0 chips were used. In total 37 arrays were analyzed. The microarray data are accessible through the Gene Expression Omnibus (GEO; GSE12466) [19,20].

### Data and statistical analysis

The processing and primary data analysis was performed in DNA-Chip Analyzer (dChip) [21]. In short, the invariant set normalization method was used [22]. Thereafter, model-based expression values were calculated according to the perfect match (PM)-only model. The criterion for fold-change analysis was set to ≥ 2-fold between groups. Signal values were then used in further statistical analysis steps such as paired and unpaired Student t-test in Excel. Some comparisons were performed using the chi square (χ^2^) test. Immunoglobulin and histocompatibility transcripts were excluded from the tables, since changes in their expression may be secondary to events unrelated to Itk-deficiency. Also the Xist and Tsix transcripts, X-chromosome encoded and unique to females, as well as Y-specific mRNAs were removed owing to that we used mixed sexes of mice in the experiments. One probe set that corresponded to Itk (1456836_at) was found to also be complementary to an EST gene (recognized at Ensembl) [23] and was therefore only included in the Additional material. We manually annotated a group of genes on the basis of prior knowledge about their role in the immune system. The 900 probe-sets list of differentially expressed transcripts between unstimulated Itk^-/- ^and Wt T-cells was used for this purpose. The classification resulted in 14 subgroups.

### Validation of differentially expressed genes using quantitative RT-PCR

Total RNA (100 ng) was reversed-transcribed into cDNA with AMV reverse transcriptase using random hexamer primers (Roche Applied Science, IN, USA). TaqMan Gene Expression Assays from Applied Biosystems were used to confirm the microarray data and it was done as previously described [24]. The validated genes were *Klrg1 *(Mm00516879_m1), *Klra3 *(Mm01702813_m1) and *Klra7 *(Mm01183384_m1). 18S rRNA was used as endogenous control.

### Chromatin-immunoprecipitation for detection of NFATc1-binding

Whole splenic and thymic cells were used for chromatin-immunoprecipitation (ChIP) assay. The cells were lysed by ammoniumchloride solution to remove erythrocytes, counted and divided into three groups each. One group was untreated, while the other two were treated with anti-CD3ε(1 μg/ml) with or without pre-treatment of CsA (1 μg/ml) for 1 hour. The stimulated cells were incubated for 24 hrs in 37°C with 5% CO_2_. The protocol for ChIP was described by Yu *et al*. [25] with the following modifications. After sonication and centrifugation, lysates were incubated with 1 μg of polyclonal anti-NFATc1 (K-18) antibody (sc-1149-R, Santa Cruz Biotechnology, Inc.) or rabbit normal Ig overnight at 4°C with rotation. Identification of targets was done by PCR using primers for the genes corresponding to *IL2, IL7R, Schlafen1, Bub1, Ctla2a and Ctla2b *(Additional file 2).

## Results

### Transcriptional changes in the absence of Itk in unstimulated CD3^+ ^T-cells

In order to survey Itk-dependent transcriptional signatures we initially conducted microarray analysis on MACS-sorted CD3^+ ^primary, unstimulated T-cells from Itk^-/- ^and Wt mice. The number of probe-sets changing ≥ 2-fold in Itk^-/- ^compared to Wt samples was 900 (2% of the total number of probe-sets on the MOE 430 2.0 chips), which is equivalent to 56% up- and 44% down-regulated probe-sets (33% with p < 0.05). From the 900-list we show the 60 most significantly up- and down-regulated transcripts in Itk-deficiency (Table 1). Most up-regulated were the killer cell lectin-like receptors Klra3 and Klra8, followed by granzyme M. Oligoadenylate synthetase-like 2 (Oasl2) was the most down-regulated transcript, next was actinin alpha 2 (Actn2). Furthermore, from the 900 probe-sets we have manually extracted 106 immune response-related genes and divided them into 14 different subgroups (Table 2). Of the 106 genes 10% were Klrs and 8.5% encoded transcription factors (Table 3). Additional file 3 shows the individual genes in each category.

**Table 1:** The 60 most up- and down-regulated transcripts in Itk-deficiency (unstimulated cells)

**Probe set**	**Gene Symbol, Gene Title**	**Itk^-/- ^vs Wt**	**t-test**
1453196_a_at	Oasl2, 2'-5' oligoadenylate synthetase-like 2	-7.91	0.008
1448327_at	Actn2, actinin alpha 2	-3.97	0.016
1437445_at	Trpm1, transient receptor potential cation channel, subfamily M, member 1	-3.72	0.051
1445450_x_at	A530021J07Rik	-3.71	0.012
1421234_at	Hnf1a, HNF1 homeobox A	-3.45	0.044
1448485_at	Ggt1, gamma-glutamyltransferase 1	-3.39	0.005
1445194_at	Cnksr2, connector enhancer of kinase suppressor of Ras 2	-3.31	0.034
1418545_at	Wasf1, WASP family 1	-3.01	0.016
1451548_at	Upp2, uridine phosphorylase 2	-2.91	0.049
1434722_at	Ampd1, adenosine monophosphate deaminase 1	-2.85	0.004
1436836_x_at	Cnn3, calponin 3, acidic	-2.79	0.005
1429274_at	2310010M24Rik	-2.46	0.001
1455442_at	Slc6a19, solute carrier family 6 member 19	-2.34	0.005
1432383_a_at	Armc9, armadillo repeat containing 9	-2.29	0.020
1417928_at	Pdlim4, PDZ and LIM domain 4	-2.28	0.055
1444801_at	2900041M22Rik	-2.27	0.029
1443570_at	Cops3, COP9 (constitutive photomorphogenic) homolog, subunit 3	-2.25	0.005
1421895_at	Eif2s3x, eukaryotic translation initiation factor 2, subunit 3	-2.25	0.013
1418055_at	Neurod4, neurogenic differentiation 4	-2.23	0.020
1453009_at	*Gene name not assigned for this probe set*	-2.22	0.014
1429350_at	Eid3, EP300 interacting inhibitor of differentiation 3	-2.21	0.039
1420877_at	Sept6, septin 6	-2.17	0.001
1438825_at	Calm3, Calmodulin 3	-2.16	0.022
1434915_s_at	Lrrc19, leucine rich repeat containing 19	-2.12	0.041
1436103_at	Rab3ip, RAB3A interacting protein	-2.12	0.006
1456751_x_at	A530021J07Rik	-2.12	0.000
1439254_at	*Gene name not assigned for this probe set*	-2.11	0.037
1418003_at	1190002H23Rik	-2.11	0.037
1449634_a_at	Anks1b, ankyrin repeat and sterile alpha motif domain containing 1B	-2.09	0.047
1418990_at	Ms4a4d, membrane-spanning 4-domains, subfamily A, member 4D	-2.09	0.031
1421182_at	Clec1b, C-type lectin domain family 1, member b	3.7	0.038
1424842_a_at	Arhgap24, Rho GTPase activating protein 24	3.74	0.014
1418340_at	Fcer1g, Fc receptor, IgE, high affinity I, gamma polypeptide	3.76	0.016
1444214_at	Tubb1, tubulin, beta 1	3.79	0.044
1452666_a_at	Tmcc2, transmembrane and coiled-coil domains 2	3.88	0.033
1457001_at	Cenpk, centromere protein K	3.9	0.004
1449340_at	Sostdc1, sclerostin domain containing 1	3.91	0.014
1434115_at	Cdh13, cadherin 13	3.95	0.054
1434955_at	March1, membrane-associated ring finger (C3HC4) 1	4.04	0.000
1439397_at	Fmn1, formin 1	4.06	0.026
1448749_at	Plek, pleckstrin	4.13	0.010
1426171_x_at	Klra7, killer cell lectin-like receptor, subfamily A, member 7	4.14	0.003
1436778_at	Cybb, cytochrome b-245, beta polypeptide	4.15	0.012
1448025_at	Sirpb1, signal-regulatory protein beta 1	4.2	0.046
1420789_at	Klra5, killer cell lectin-like receptor, subfamily A, member 5	4.22	0.018
1441887_x_at	EG622976	4.26	0.017
1438553_x_at	*Gene name not assigned for this probe set*	4.28	0.011
1417765_a_at	Amy1, amylase 1, salivary	4.29	0.016
1451263_a_at	Fabp4, fatty acid binding protein 4	4.32	0.020
1427866_x_at	*Gene name not assigned for this probe set*	4.43	0.037
1454200_at	Zeb2, zinc finger E-box binding homeobox 2	4.57	0.022
1420492_s_at	Smr3a, submaxillary gland androgen regulated protein 3A	4.79	0.006
1427503_at	AI324046	4.85	0.024
1437463_x_at	Tgfbi, transforming growth factor, beta induced	5.04	0.005
1419348_at	Psp, parotid secretory protein	5.11	0.019
1419874_x_at	Zbtb16, zinc finger and BTB domain containing 16	5.46	0.005
1442025_a_at	*Gene name not assigned for this probe set*	5.48	0.002
1449501_a_at	Gzmm, granzyme M	6.39	0.013
1425436_x_at	Klra3, killer cell lectin-like receptor, subfamily A, member 3	9.92	0.000
1425417_x_at	Klra8, killer cell lectin-like receptor, subfamily A, member 8	35.69	0.000

The down-regulated transcripts are shown with "-"

**Table 2:** Groups of genes expressed in the immune response group

**Immune response groups**	**Number of genes involved**	**Immune response groups**	**Number of genes involved**
Chemokine receptors	5	Interleukins	4
Chemokines	8	Intracellular signaling components	7
Colony stimulating factor receptors	4	Killer cell lectin-like receptors	11
Fc receptors	5	Miscellaneous	24
Granzymes	4	Other surface antigens with CD-designation	17
Interferon-related genes	3	Toll-like receptors	2
Interleukin receptors	4	Transcription factors	9

**Table 3:** The genes found in Killer cell lectin-like receptor and transcription factor groups from Table 2

**Killer cell lectin-like receptors**		
**Probe set**	**Gene title**	**Itk^-/- ^vs Wt**
1458642_at	killer cell lectin-like receptor family E member 1 (NKG2I)	2.6
1451664_x_at	killer cell lectin-like receptor subfamily A, member 12 (Ly49L)	2.13
1422065_at	killer cell lectin-like receptor subfamily B member 1B (Ly55B/Ly55D)	3.16
1425005_at	killer cell lectin-like receptor subfamily C, member 1 (NKG2A/2B)	2.13
1420790_x_at	killer cell lectin-like receptor, subfamily A, member 16 (Ly49P)	-2.62
1426127_x_at	killer cell lectin-like receptor, subfamily A, member 18 (Ly49R)	2.99
1426140_x_at	killer cell lectin-like receptor, subfamily A, member 19 (Ly49S)	2.73
1425436_x_at	killer cell lectin-like receptor, subfamily A, member 3 (Ly49C)	9.92
1420789_at	killer cell lectin-like receptor, subfamily A, member 5 (Ly49E)	4.22
1426171_x_at	killer cell lectin-like receptor, subfamily A, member 7 (Ly49G)	4.14
1425417_x_at	killer cell lectin-like receptor, subfamily A, member 8 (Ly49H)	35.69
		
**Transcription factors**		
**Probe set**	**Gene title**	**Itk^-/- ^vs Wt**
1416916_at	E74-like factor 3	2.97
1457441_at	early B-cell factor 1	*
1416301_a_at	early B-cell factor 1	*
1435172_at	eomesodermin homolog (Xenopus laevis)	2.56
1426001_at	eomesodermin homolog (Xenopus laevis)	3.07
1421303_at	IKAROS family zinc finger 1	-2.2
1422537_a_at	inhibitor of DNA binding 2	2.03
1447640_s_at	pre B-cell leukemia transcription factor 3	2.05
1460407_at	Spi-B transcription factor (Spi-1/PU.1 related)	2.38
1429427_s_at	transcription factor 7-like 2, T-cell specific, HMG-box	2.41
1419874_x_at	zinc finger and BTB domain containing 16	5.46

* Early B-cell factor 1 showed variable expression changes for different probe sets

The down-regulated transcripts are shown with "-"

### Transcriptional changes in the absence of Itk in stimulated CD3^+ ^T-cells

**Figure 1:**
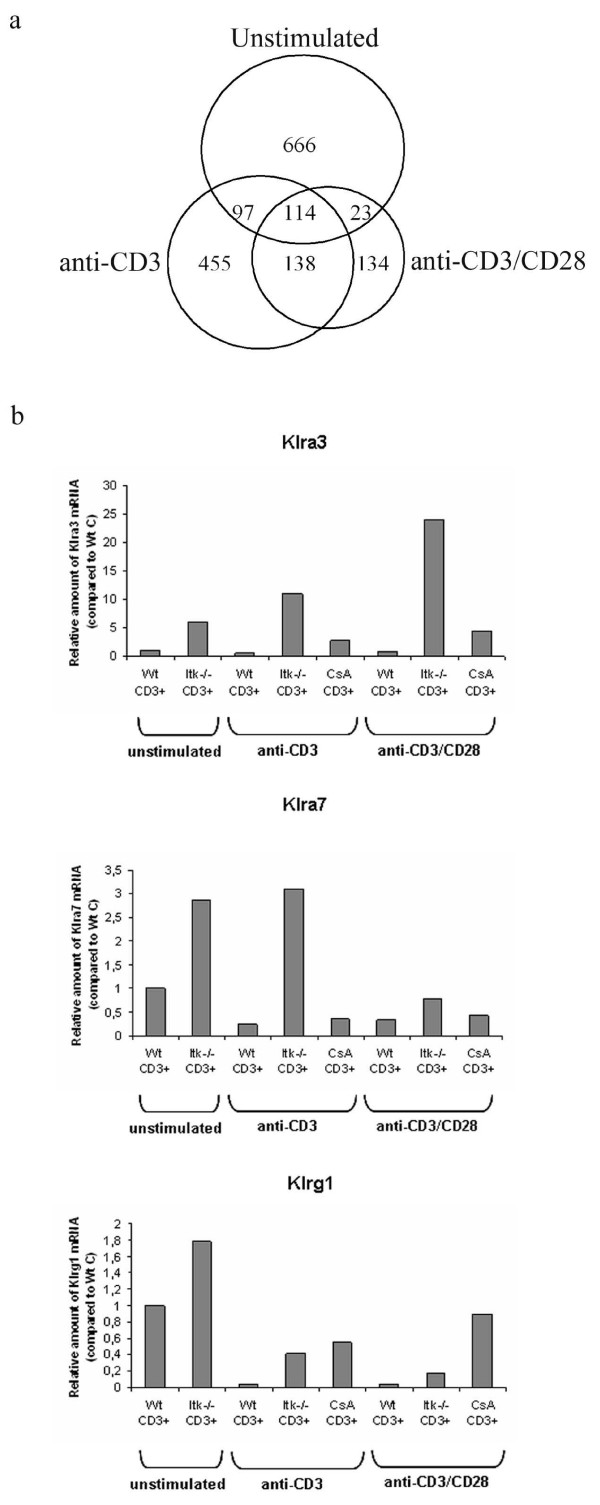
**The number of differentially expressed probe-sets in Itk-deficiency**. **a**. Venn diagram showing overlapping probe-sets in CD3^+ ^Itk-defective T-cells, unstimulated (upper), anti-CD3- (left) and anti-CD3/CD28-stimulated (right). All the comparisons were made against Wt and with the criterion ≥ 2-fold. **b**. Quantitative RT-PCR confirms up-regulated expression of Klra3, Klra7 and Klrg1 in Itk-deficiency. The bar charts show relative amount of Klr mRNA compared to unstimulated Wt CD3^+ ^T-cells (Wt C).

Stimulating the Itk^-/- ^and Wt T-cells with anti-CD3 resulted in 804 differentially expressed probe-sets in Itk-deficiency (74% up- and 26% down-regulated, 68% with p < 0.05), while after anti-CD3/CD28-stimulation the number was reduced to 409 (78% up- and 22% down-regulated; 58% with p < 0.05) as depicted in Figure 1a. Between CD3- and CD3/CD28-stimulations, the overlap was 252 probe-sets (see Table 4 for a list of the 60 most up- and down-regulated transcripts). We show there that Itk was the most down-regulated transcript in Itk-deficiency, followed by Crabp2, which encodes cellular retinoic acid binding protein 2. This is a 15 kD regulator of retinoic acid signaling recently reported to be differentially expressed in acute lymphoblastic leukaemia [26]. Other down-regulated transcripts were IL-2 and IL-3.

**Table 4:** The 60 most up- and down-regulated transcripts in Itk-deficiency after anti-CD3- (1) and anti-CD3/CD28-stimulation (2)

**Probe set**	**Gene Symbol, Gene Title**	**Itk^-/- ^vs Wt (1)**	**t-test**	**Itk^-/- ^vs Wt (2)**	**t-test**
1457120_at	Itk, IL2-inducible T-cell kinase	-6.96	0.007	-6.63	0.009
1451191_at	Crabp2, cellular retinoic acid binding protein II	-5.31	0.013	-2.79	0.054
1449990_at	Il2///LOC630222, interleukin 2	-3.89	0.002	-5.17	0.030
1436194_at	Prelid2, PRELI domain containing 2	-3.54	0.036	-2.42	0.077
1437935_at	4930486G11Rik, RIKEN cDNA	-3.44	0.032	-3.16	0.079
1439995_at	Nhedc2, Na+/H+ exchanger domain containing 2	-2.92	0.021	-2.71	0.028
1441971_at	*Gene name not assigned for this probe set*	-2.91	0.050	-2.88	0.082
1426243_at	Cth, cystathionase	-2.9	0.000	-2.8	0.072
1438380_at	Ddx47, DEAD box polypeptide 47	-2.69	0.002	2.03	0.300
1450566_at	Il3, interleukin 3	-2.68	0.031	-2.81	0.005
1420843_at	Ptprf, protein tyrosine phosphatase, receptor type,	-2.49	0.027	-2.07	0.222
1448788_at	Cd200, Cd200 antigen	-2.47	0.014	-2.43	0.011
1427049_s_at	Smo, smoothened homolog (Drosophila)	-2.46	0.000	-2.45	0.015
1422070_at	Adh4, alcohol dehydrogenase 4 (class II)	-2.33	0.015	-2.26	0.157
1456226_x_at	Ddr1, discoidin domain receptor family, member 1	-2.29	0.014	-2.91	0.038
1419136_at	Akr1c18, aldo-keto reductase family 1, member C18	-2.09	0.052	2.41	0.008
1433571_at	Serinc5, serine incorporator 5	-2	0.011	-2.05	0.125
1425832_a_at	Cxcr6, chemokine (C-X-C motif) receptor 6	5.34	0.000	3.27	0.085
1437463_x_at	Tgfbi, transforming growth factor, beta induced	5.36	0.045	2.4	0.012
1421802_at	Ear1, eosinophil-associated, ribonuclease A family, member 1	5.38	0.017	2.8	0.000
1448620_at	Fcgr3, Fc receptor, IgG, low affinity III	5.57	0.036	3.72	0.004
1438855_x_at	Tnfaip2, tumor necrosis factor, alpha-induced protein 2	5.6	0.0034	3.12	0.000
1450009_at	Ltf, lactotransferrin	5.68	0.080	2.03	0.034
1416514_a_at	Fscn1, fascin homolog 1	5.7	0.000	3.69	0.096
1451948_at	Gm1409, gene model 1409	5.81	0.002	3.45	0.136
1451675_a_at	Alas2, aminolevulinic acid synthase 2, erythroid	5.84	0.003	2.92	0.096
1420330_at	Clec4e, C-type lectin domain family 4, member e	5.89	0.014	3.15	0.091
1420699_at	Clec7a, C-type lectin domain family 7, member a	5.98	0.014	4.05	0.004
1427747_a_at	Lcn2, lipocalin 2	5.98	0.056	2.23	0.011
1427503_at	AI324046, expressed sequence AI324046	6.33	0.005	3.48	0.055
1419082_at	Serpinb2, serine (or cysteine) peptidase inhibitor, clade B, member 2	6.34	0.001	2.51	0.125
1419627_s_at	Clec4n, C-type lectin domain family 4, member n	6.36	0.021	2.6	0.0129
1448213_at	Anxa1, annexin A1	6.43	0.069	3.07	0.029
1419874_x_at	Zbtb16, zinc finger and BTB domain containing 16	6.62	0.032	5.3	0.001
1417898_a_at	Gzma, granzyme A	6.71	0.009	5.59	0.001
1419598_at	Ms4a6d, membrane-spanning 4-domains, subfamily A, member 6D	6.73	0.005	3.27	0.083
1429889_at	Faim3, Fas apoptotic inhibitory molecule 3	6.73	0.047	3.01	0.041
1415904_at	Lpl, lipoprotein lipase	6.76	0.054	4.1	0.026
1449254_at	Spp1, secreted phosphoprotein 1	6.8	0.028	3.79	0.020
1427910_at	Cst6, cystatin E/M	7.1	0.023	4.17	7.43E-06
1438553_x_at	*Gene name not assigned for this probe set*	7.11	0.011	4.63	0.055
1434150_a_at	Mettl7a///Ubie, methyltransferase like 7A	7.14	0.000	4.04	0.185
1442025_a_at	AI467657, expressed sequence AI467657	7.48	0.041	6.52	0.000
1436778_at	Cybb, cytochrome b-245, beta polypeptide	7.77	0.077	4.03	0.003
1439426_x_at	Lyz, lysozyme	7.78	0.006	2.98	0.011
1449846_at	Ear2///Ear3, eosinophil-associated, ribonuclease A family, member 2	8.62	0.008	3.54	0.031
1434194_at	Mtap2, microtubule-associated protein 2	8.73	0.035	5.78	0.000
1427866_x_at	Beta globin	9.29	0.001	5.44	0.074
1450912_at	Ms4a1, membrane-spanning 4-domains, subfamily A, member 1	9.4	0.087	4.31	0.036
1422873_at	Prg2, proteoglycan 2, bone marrow	9.81	0.028	4.19	0.026
1419764_at	Chi3l3, chitinase 3-like 3	10.51	0.070	4.1	0.004
1422411_s_at	Ear1///Ear12///Ear2///Ear3, eosinophil-associated, ribonuclease A family, member 1	10.54	0.010	4.61	0.000
1418722_at	Ngp, neutrophilic granule protein	11.39	0.031	3.74	0.006
1450989_at	LOC100047300///Tdgf1, teratocarcinoma-derived growth factor	11.64	0.009	2.89	0.076
1419394_s_at	S100a8, S100 calcium binding protein A8	12.08	0.071	4.6	0.034
1425436_x_at	Klra3, killer cell lectin-like receptor, subfamily A, member 3	12.34	0.002	14.75	0.000
1415897_a_at	Mgst1, microsomal glutathione S-transferase 1	12.62	0.082	3.77	0.006
1448756_at	S100a9, S100 calcium binding protein A9	12.97	0.077	5.15	0.010
1426171_x_at	Klra7, killer cell lectin-like receptor, subfamily A, member 7	14.08	0.027	14.02	0.015
1425417_x_at	Klra8, killer cell lectin-like receptor, subfamily A, member 8	21.07	0.026	17.99	0.000

The down-regulated transcripts are shown with "-"

Stimulation affected the majority of the transcripts in the same direction as observed in unstimulated cells (p < 10^-6^) (only in 5/252 cases the CD3- or CD3/CD28-stimulations showed opposite fold-changes; Additional file 4). We show there that the most induced mRNA was Klra8 (Ly49H) (21-fold-change upon anti-CD3-stimulation). Other up-regulated Klrs were Klra3 (Ly49C), Klra5 (Ly49E), Klra7 (Ly49G), Klra19 (Ly49S), Klrc1 (NKG2A/2B), Klrd1 (CD94), Klre1 (NKG2I) and Klrg1 (2P1-Ag). By quantitative RT-PCR we confirmed the up-regulated expression of Klrg1, Klra3 and Klra7 in Itk-defective samples (Fig. 1b). Two transcription factors, inhibitor of DNA binding 2 (Id2) and eomesodermin, were also found up-regulated. Thus, in CD3^+ ^cells, differential transcriptional signatures between Wt and Itk-deficient cells were more pronounced in unstimulated when compared to activated cells.

**Figure 2:**
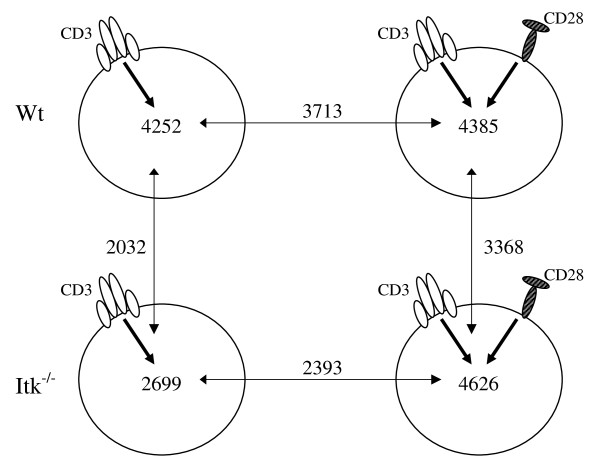
**The amount of differentially expressed probe-sets in Wt and Itk-defective CD3^+ ^T-cells following stimulation**. The upper panel represents the Wt T-cells and the lower the Itk-defective T-cells. The left panel symbolizes the anti-CD3-stimulation and the right panel the anti-CD3/CD28-stimulation. All the stimulations were compared to the untreated condition. The arrows denote the number of overlapping probe-sets.

We continued to analyze the activation-dependent signatures in Wt and Itk^-/- ^T-cells separately. The number of probe-sets changing ≥ 2-fold after anti-CD3-stimulation (compared to the unstimulated state) in the Wt samples was 4252, and the corresponding number after co-stimulation was 4385 (Figure 2). The overlap between the two stimulations was 3713 (87% and 85%, respectively; Additional file 5). However, the differences were significantly more pronounced in anti-CD3 versus anti-CD3/CD28 activated cells in the Itk-defective group, with only 50% of the transcripts in the co-stimulated group overlapping with the CD3-stimulated (p < 10^-6^) (Additional file 6). Thus, co-stimulation had much greater effect on Itk-deficient than on Wt cells. We further examined some of the immune response-related genes previously mentioned. Ten members of the Klr family were up-regulated in unstimulated Itk-defective compared to Wt samples (Table 3), while after stimulation the majority of Klrs were down-regulated in both Wt and Itk-defective T-cells. Down-regulation of Klrs were also reported for human cells from healthy individuals in a recent paper by Wang *et al*., where primary human T-cells were analyzed after anti-CD3/CD28-stimulation [27]. With respect to cytokine expression, IL-2 and IL-6 were found up-regulated upon anti-CD3-stimulation in both Wt and Itk-deficient samples when compared to the corresponding unstimulated cells. In contrast, IL-16 and IL-18 were down-regulated (Additional files 5 and 6). Two cytokines, whose expression was only altered in Itk-deficient cells upon anti-CD3-stimulation, were IL-10 (up-regulated) and IL-33 (down-regulated) (data not shown). IL-33 is a novel IL-1 family cytokine, IL1F11/IL-33, playing an important role in eosinophil-mediated inflammation [28]. Interestingly, Itk-deficient mice have previously been shown to have reduced lung inflammation, eosinophil infiltration and mucous production after induction of allergic asthma [29]. No other cytokines were differentially expressed. In the stimulated Wt samples we also observed altered expression of several transcription factors such as Zbtb16 (encoding the transcriptional regulator PLZF), Id2 and Spi-C, while in Itk-defective cells we found Zbtb16, Spi-C and Id3 to be differentially expressed upon stimulation.

### CD4^+ ^and CD8^+ ^T-cell signatures in Itk-deficiency

As T-cells can be divided into CD4^+ ^and CD8^+ ^subsets, and as these subsets have very distinct functions and gene regulations, we examined Itk-deficiency in MACS purified CD4^+ ^and CD8^+ ^Wt and Itk-deficient T-cells. The Itk-deficient CD4^+ ^and CD8^+ ^T-cells are known from previous studies to be of a memory-like phenotype, characterized by the markers CD44 and CD122 [9-11,13]. Since CD122 expression is enhanced by the transcription factors eomesodermin and T-box 21 (T-bet), we looked for their expression in our data. Eomesodermin was previously reported to be up-regulated in Itk-deficient T-cells [9] and we found the expression of eomesodermin much higher in the CD8^+ ^T-cell population compared to CD4^+ ^in unstimulated condition. The same was also seen with T-bet. Taken together, the observed expression pattern of eomesodermin and T-bet is in agreement with previously published studies and thus validates our microarray data. Moreover, our analysis also includes new knowledge related to these transcripts, namely how they respond to activation of T-cells as well as the effect of CsA (Additional file 7).

**Figure 3:**
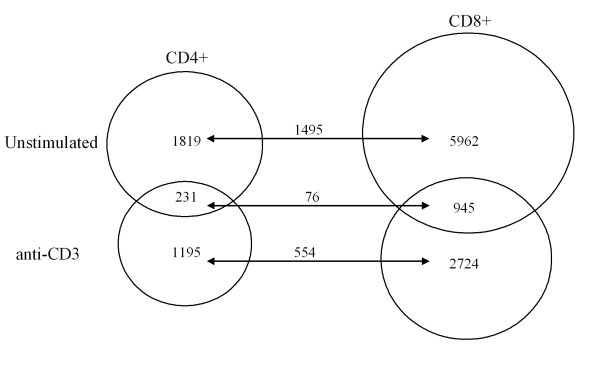
**Overlapping probe-sets in unstimulated and anti-CD3-stimulated Itk-deficient CD4^+ ^(left) and CD8^+ ^(right) T-cell populations**. Each circle is a comparison between Itk-defective and Wt samples. All the comparisons were made with the criterion ≥ 2-fold.

Both CD4^+ ^and CD8^+ ^T-cell subsets in Itk-deficient mice have been shown to differ in phenotype compared to the Wt mice. In the absence of Itk, a higher percentage of each subset expresses surface markers, typical for memory phenotype cells, such as CD44^hi ^and CD122^hi ^[9,10,13]. We sought to determine whether this was also reflected by their transcriptomes. The number of transcripts differentially expressed between unstimulated Itk^-/- ^and Wt in the CD4^+ ^population was 2050, while in the CD8^+ ^population the number was higher (n = 6907). The 60 most up- and down-regulated transcripts from each subset are shown in Tables 5 and 6. Among those are genes already mentioned, e.g. eomesodermin, Klra3 and 8, T-bet and Granzyme M. In these groups we also found Zbtb16. Interestingly, Zbtb16 was 12-fold up-regulated in CD4^+ ^cells and 45-fold down-regulated in CD8^+ ^cells, also suggesting a highly efficient separation of the two subsets. Based on these findings PLZF was selected for further studies presented elsewhere [30]. The most pronounced changes were seen in the CD8^+ ^population (Table 6), with 69-fold down-regulation of Clca1, which is a calcium-activated chloride-channel, of importance in airway epithelial cells. Two up-regulated transcripts were the PTB-domain containing MAP-kinase regulator Dok5 (29-fold) and α-tubulin (30-fold), whose expression in T-cells, to our knowledge, was not previously reported. After anti-CD3-stimulation we found the number of differentially expressed transcripts reduced in both subsets, approximately 30% and 47% fewer probe-sets in CD4^+ ^and CD8^+^, respectively. The overlapping probe-sets between the unstimulated and the anti-CD3-stimulated conditions are shown in Figure 3. 82% of the transcripts in the CD4^+ ^subset were also found in the CD8^+ ^population in unstimulated cells. The percentage of overlapping transcripts decreased with stimulation.

**Table 5:** The 60 most up- and down-regulated transcripts in Itk-defective CD4^+ ^T-cells (unstimulated cells)

**Probe set**	**Gene Symbol, Gene Title**	**Itk^-/- ^vs Wt**	**t-test**
1436386_x_at	OTTMUSG00000010671	-7.51	0.012
1444708_at	Tmem29, transmembrane protein 29	-5	0.014
1434418_at	Lass6, LAG1 homolog, ceramide synthase 6	-4.93	0.001
1438354_x_at	Cnn3, Calponin 3, acidic	-4.81	0.030
1430988_at	2810407C02Rik	-3.84	0.028
1430827_a_at	Ptk2, PTK2 protein tyrosine kinase 2	-3.68	0.002
1458977_at	A530021J07Rik	-3.36	0.006
1439778_at	Cables1, Cdk5 and Abl enzyme substrate 1	-3.26	0.018
1427675_at	V1ra2, vomeronasal 1 receptor, A2	-3.22	0.041
1448338_at	Pgcp, plasma glutamate carboxypeptidase	-3.16	0.001
1458945_at	AU015148	-3.16	0.038
1421507_at	Olfr78, olfactory receptor 78	-3.07	0.021
1457120_at	Itk, IL2-inducible T-cell kinase	-2.96	0.002
1456178_at	Bambi-ps1, BMP and activin membrane-bound inhibitor, pseudogene (Xenopus laevis)	-2.94	0.000
1455907_x_at	Phox2b, paired-like homeobox 2b	-2.88	0.050
1452474_a_at	Art3, ADP-ribosyltransferase 3	-2.86	0.026
1459508_at	C85600	-2.79	0.007
1440761_at	4833422C13Rik	-2.77	0.052
1446412_at	*Gene name not assigned for this probe set*	-2.71	0.012
1441221_at	*Gene name not assigned for this probe set*	-2.7	0.019
1427632_x_at	Cd55, CD55 antigen	-2.65	0.042
1439181_at	BC043301	-2.63	0.032
1434473_at	Slc16a5, solute carrier family 16 (monocarboxylic acid transporters), member 5	-2.59	0.001
1448002_x_at	2610001J05Rik	-2.57	0.018
1455425_at	BB001228	-2.57	0.016
1419620_at	Pttg1, pituitary tumor-transforming 1	-2.53	0.000
1453009_at	*Gene name not assigned for this probe set*	-2.39	0.000
1455740_at	Hnrnpa1, heterogeneous nuclear ribonucleoprotein A1	-2.31	0.048
1429413_at	Cpm, carboxypeptidase M	-2.28	0.003
1416441_at	Pgcp, plasma glutamate carboxypeptidase	3.12	0.005
1425216_at	Ffar2, free fatty acid receptor 2	3.13	0.036
1448471_a_at	Ctla2a, cytotoxic T lymphocyte-associated protein 2 alpha	3.13	0.006
1450334_at	Il21, interleukin 21	3.13	0.013
1423091_a_at	Gpm6b, glycoprotein m6b	3.21	0.032
1435339_at	Kctd15, potassium channel tetramerisation domain containing 15	3.24	0.044
1428197_at	Tspan9, tetraspanin 9	3.37	0.001
1449036_at	Rnf128, ring finger protein 128	3.44	0.002
1449361_at	Tbx21, T-box 21	3.5	0.012
1447839_x_at	Adm, adrenomedullin	3.62	0.031
1418318_at	Rnf128, ring finger protein 128	3.77	0.021
1419647_a_at	Ier3, immediate early response 3	3.78	0.005
1448961_at	Plscr2, phospholipid scramblase 2	4.06	0.012
1449280_at	Esm1, endothelial cell-specific molecule 1	4.15	0.013
1427445_a_at	Ttn, titin	4.32	0.006
1425471_x_at	*Gene name not assigned for this probe set*	4.33	0.050
1438553_x_at	*Gene name not assigned for this probe set*	4.35	0.019
1423231_at	Nrgn, neurogranin	4.66	0.002
1416846_a_at	Pdzrn3, PDZ domain containing RING finger 3	4.69	0.002
1430946_at	2600014E21Rik	4.82	0.031
1426001_at	Eomes, eomesodermin homolog (Xenopus laevis)	5.43	0.024
1422280_at	Gzmk, granzyme K	5.6	0.002
1427608_a_at	Tcrg-V1, T-cell receptor gamma, variable 1	5.62	0.044
1434194_at	Mtap2, microtubule-associated protein 2	5.71	0.040
1434115_at	Cdh13, cadherin 13	6.15	0.047
1455435_s_at	Chdh, choline dehydrogenase	6.54	0.041
1449864_at	Il4, interleukin 4	6.77	0.031
1424011_at	Aqp9, aquaporin 9	7.23	0.011
1420678_a_at	Il17rb, interleukin 17 receptor B	9.84	0.029
1442025_a_at	*Gene name not assigned for this probe set*	11.69	0.008
1419874_x_at	Zbtb16, zinc finger and BTB domain containing 16	12.07	0.009

The down-regulated transcripts are shown with "-"

**Table 6:** The 60 most up- and down-regulated transcripts in Itk-defective CD8^+ ^T-cells (unstimulated cells)

**Probe set**	**Gene Symbol, Gene Title**	**Itk^-/- ^vs Wt**
1417852_x_at	Clca1, chloride channel calcium activated 1	-68.71
1419874_x_at	Zbtb16, zinc finger and BTB domain containing 16	-45.13
1436759_x_at	Cnn3, calponin 3, acidic	-39.91
1454869_at	Wdr40b, WD repeat domain 40B	-34.02
1427054_s_at	Abi3bp, ABI gene family, member 3 (NESH) binding protein	-31.51
1437992_x_at	Gja1, gap junction protein, alpha 1	-25.36
1416203_at	Aqp1, aquaporin 1	-24.68
1437279_x_at	Sdc1, syndecan 1	-24.33
1448182_a_at	Cd24a, CD24a antigen	-21.41
1456956_at	Ikzf2, IKAROS family zinc finger 2	-19.8
1442025_a_at	*Gene name not assigned for this probe set*	-19.78
1439422_a_at	C1qdc2, C1q domain containing 2	-19.69
1454086_a_at	Lmo2, LIM domain only 2	-18.62
1416330_at	Cd81, CD81 antigen	-17.08
1451867_x_at	Arhgap6, Rho GTPase activating protein 6	-17.08
1456060_at	Maf, avian musculoaponeurotic fibrosarcoma (v-maf) AS42 oncogene homolog	-16.84
1419014_at	Rhag, Rhesus blood group-associated A glycoprotein	-16.79
1416193_at	Car1, carbonic anhydrase 1	-16.49
1450744_at	Ell2, elongation factor RNA polymerase II 2	-14.83
1456147_at	St8sia6, ST8 alpha-N-acetyl-neuraminide alpha-2,8-sialyltransferase 6	-14.78
1456475_s_at	Prkar2b, protein kinase, cAMP dependent regulatory, type II beta	-14.57
1460431_at	Gcnt1, glucosaminyl (N-acetyl) transferase 1, core 2	-14.03
1437171_x_at	Gsn, gelsolin	-13.64
1417777_at	Ltb4dh, leukotriene B4 12-hydroxydehydrogenase	-13.54
1437935_at	4930486G11Rik	-13.51
1434499_a_at	Ldhb, lactate dehydrogenase B	-13.44
1450333_a_at	Gata2, GATA binding protein 2	-13.33
1423569_at	Gatm, glycine amidinotransferase (L-arginine:glycine amidinotransferase)	-12.94
1435884_at	Itsn1, intersectin 1 (SH3 domain protein 1A)	-12.72
1425145_at	Il1rl1, interleukin 1 receptor-like 1	-12.6
1449313_at	Klk1b5, kallikrein 1-related peptidase b5	13.38
1454106_a_at	Cxxc1, CXXC finger 1 (PHD domain)	13.41
1442639_at	*Gene name not assigned for this probe set*	13.76
1443006_at	*Gene name not assigned for this probe set*	13.93
1433449_at	Snx32, sorting nexin 32	14.78
1423603_at	Zfpm1, zinc finger protein, multitype 1	14.98
1441770_at	Ppat, phosphoribosyl pyrophosphate amidotransferase	15.29
1420343_at	Gzmd, granzyme D	15.42
1445596_at	*Gene name not assigned for this probe set*	15.54
1452985_at	Uaca, uveal autoantigen with coiled-coil domains and ankyrin repeats	16.55
1420233_at	*Gene name not assigned for this probe set*	16.79
1423020_at	*Gene name not assigned for this probe set*	17.39
1454481_at	Mif, macrophage migration inhibitory factor	17.64
1427426_at	Kcnq5, potassium voltage-gated channel, subfamily Q, member 5	18.06
1424698_s_at	Gca, grancalcin	18.46
1447574_s_at	Slc32a1, Solute carrier family 32 (GABA vesicular transporter), member 1	18.73
1431854_a_at	4930452B06Rik	18.94
1460267_at	Dmrt3, doublesex and mab-3 related transcription factor 3	19.76
1417197_at	Wwc2, WW, C2 and coiled-coil domain containing 2	20.76
1442788_at	Afap1, actin filament associated protein 1	20.91
1447292_at	Actr1b, ARP1 actin-related protein 1 homolog B (yeast)	21.96
1431878_at	Grhl2, grainyhead-like 2 (Drosophila)	22.18
1449501_a_at	Gzmm, granzyme M (lymphocyte met-ase 1)	23.58
1436500_at	Rps24, Ribosomal protein S24	25.96
1425436_x_at	Klra3, killer cell lectin-like receptor, subfamily A, member 3	26.39
1456130_at	LOC553091	27.36
1454240_at	Nfe2l3, nuclear factor, erythroid derived 2, like 3	29.25
1422641_at	Dok5, docking protein 5	29.31
1417375_at	Tuba4a, tubulin, alpha 4A	30.36
1425417_x_at	Klra8, killer cell lectin-like receptor, subfamily A, member 8	131.81

The down-regulated transcripts are shown with "-"

The largest number of differentially expressed transcripts was observed in the unstimulated groups of Itk-deficient CD4^+ ^and CD8^+ ^T-cell subsets. By subtracting the overlapping 1495 probe-sets (Figure 3) from unstimulated Itk-deficient CD4^+ ^and CD8^+ ^cells, respectively, we characterized separate core groups of transcripts for each subset. The remaining number of probe-sets in the CD4^+ ^population was 324 (Additional file 8), while it was more than 10-fold higher in the CD8^+ ^group (Additional file 9). Interestingly, two members of the Klr family (Klrb1a (Ly55a) and Klrb1c (NK-1.1)) were present in the CD4^+^population, while four other members were found in the CD8^+ ^subset (Klra4 (Ly49D), Klra19 (Ly49S), Klrc2 (NKG2C) and Klrk1 (NKG2D)). Five Klrs were in common between unstimulated CD4^+ ^and CD8^+ ^groups, they were Klra3, a7, a8, a22 and b1b (by comparing 2050 and 6907 in Fig. 3). The differentially expressed NK/innate cell-related transcripts were not limited to cell surface markers, since RNA for the cytotoxic protein Granzyme M was strongly enriched in the Itk-deficient population, again confirming that NK- and innate cells have overlapping transcriptomes [31].

### Itk-deficiency mimics calcineurin inhibition

Tec-family kinases activate PLCγ and are therefore important regulators of Ca^2+^-mobilization and the calcineurin/NFAT pathway [5,32]. However, Tec-family kinases regulate also other signaling pathways. To investigate which of the Itk-related changes is the consequence of an impaired calcineurin/NFAT pathway, we compared the expression profiles of anti-CD3 ± CD28 stimulated Itk^-/- ^CD3^+ ^T-cells and of CsA-treated Wt T-cells. CsA specifically inhibits calcineurin and by that affects downstream signaling and the activation of the transcription factors of the NFAT-family. Based on the dependency of Itk and/or calcineurin, three groups of genes could be identified: Itk- and calcineurin-dependent (Itk/CN); Itk-dependent and calcineurin-independent (Itk/non-CN) and Itk-independent and calcineurin-dependent (non-Itk/CN).

**Figure 4:**
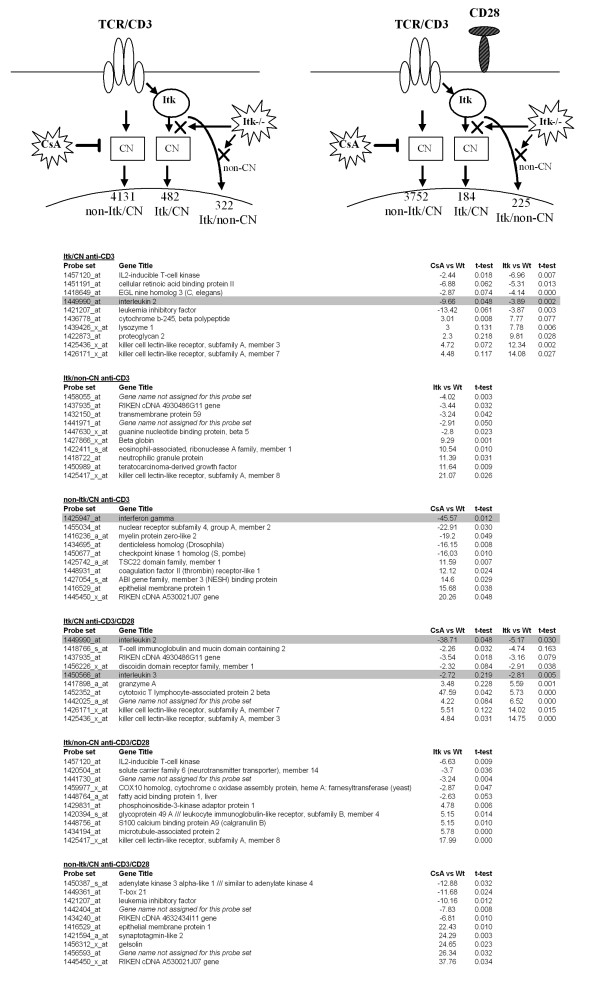
**Anti-CD3- and anti-CD3/CD28-stimulations leading to Itk- and calcineurin-dependent and -independent transcriptional signatures**. Three different groups of genes exist in each stimulation condition. The groups are Itk- and calcineurin-dependent (Itk/CN), Itk-dependent and calcineurin-independent (Itk/non-CN) and Itk-independent and calcineurin-dependent (non-Itk/CN). 113 probe-sets are overlapping between Itk/CN groups in the two stimulations. In each group the 10 most highly regulated transcripts are presented. All the chosen genes passed the t-test criterion of p < 0.05. The down-regulated transcripts are shown with "-". The transcripts in the two Itk/CN groups passed the criterion in at least one of the two comparisons. Genes previously known to be calcineurin-regulated are grey-shaded [16]. Arrows denoting signal transduction from CD28 have been omitted for clarity.

Altogether, after anti-CD3-stimulation 4613 probe-sets were differentially expressed in CsA-treated cells compared to untreated, and after co-stimulation the number was reduced by 15% to 3936. The gene numbers observed in Itk-deficient compared to Wt cells were 804 and 409 after anti-CD3- and anti-CD3/CD28-stimulation, respectively (Figure 1a). About 60% of the probe-sets that were changed ≥ 2-fold in Itk^-/- ^compared to Wt after anti-CD3-stimulation were also found in the CsA-treated samples (Figure 4, Itk/CN anti-CD3, showing the 10 most highly-regulated transcripts). In co-stimulated cells 45% of the probe-sets were the same (Figure 4, Itk/CN anti-CD3/CD28). When comparing the Itk-dependent probe-sets being calcineurin-dependent in both stimulations the overlap was 113 (Additional file 10). As expected, IL-2 was found in that group, confirming the biological relevance of our data, since IL-2 is known to be both Itk- and calcineurin-dependent [16,33]. Among other genes found in this group were *Zbtb16 *(up-regulated) and *Crabp2 *(down-regulated). Interestingly, the transcript for chemokine (C-motif) ligand 1 (Xcl1) was down-regulated in the CsA-treated cells while it showed increased expression in Itk-deficient samples. Furthermore, cytotoxic T lymphocyte-associated protein 2 alpha and beta (*Ctla2a *and *Ctla2b*) were up-regulated. Interestingly, their altered expression was more pronounced in the CsA-treated (>3 times higher) than in the Itk-deficient samples. Two granzyme-encoding genes, *Gzma *and *Gzmk*, were also found among those that were Itk- and calcineurin-dependent.

The fractions of Itk/non-CN genes (322 vs 225- for anti-CD3- and anti-CD3/CD28-stimulated T-cells, respectively) shared 95 probe-sets, corresponding to 89 transcripts (Additional file 11). Among them were three up-regulated members of the Klrs; *Klra5, Klra8 *and *Klre1*. After co-stimulation, a much smaller number of probe-sets were Itk-dependent compared to anti-CD3-stimulation only (p < 10^-6^). The transcripts being calcineurin-dependent but Itk-independent (non-Itk/CN group) were 4131 and 3752 in anti-CD3- and co-stimulated cells, respectively. It is interesting to note that CsA-treatment, but not Itk-deficiency (the non-Itk/CN anti-CD3 group), results in severely reduced transcript levels for IFNγ. Previous studies show that an immediate IFNγ release is a hallmark of the innate CD8-population [9,10].

### NFAT-binding genes that are Itk- and calcineurin- dependent

**Figure 5:**
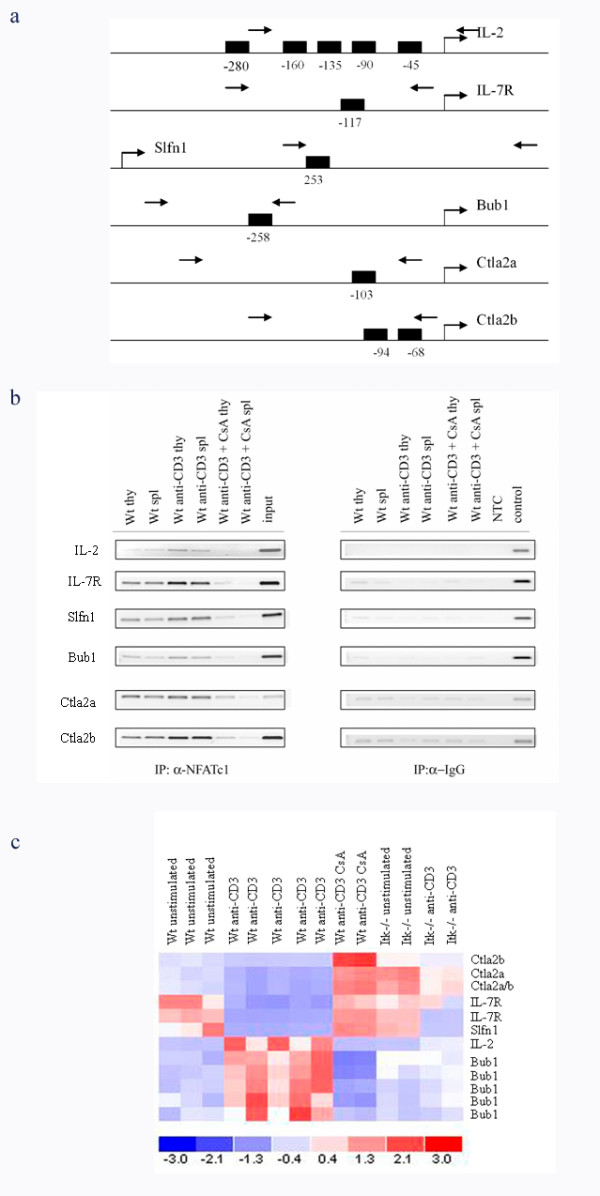
**NFAT-binding genes being Itk- and calcineurin-dependent**. **a**. Promoter regions with NFAT-binding sites in *IL2, IL7R, Bub1, Slfn1 (Schlafen1), Ctla2a and Ctla2b *genes are shown with the binding site(s) represented by black boxes. This identification was done by bioinformatic analyses of the 500 bp region upstream of each gene's transcriptional start site. This approach identified 1–2 NFAT binding sites within the promoter regions of these genes as well as upstream of the translation initiation of *Slfn1 *gene. The numbers below each box represent the position of the binding site in correlation to the transcription start. The arrows indicate forward and reverse primers. **b**. NFATc1-binding in *IL2, IL7R, Bub1, Slfn1, Ctla2a and Ctla2b *genes. CD3^+ ^T-cells were isolated from Wt thymus (thy) and spleen (spl) as described in experimental procedures. The PCR pictures were analyzed with Fluoro-S gel documentation equipment (BioRad Laboratories, CA) with a CCD camera, and further evaluated using the Quantity One software. Input; DNA before IP, NTC; no template control. **c**. Signal intensities of the six genes in Wt unstimulated, Wt anti-CD3-stimulated, Wt anti-CD3-stimulated + CsA-treated, Itk-defective unstimulated and Itk-defective anti-CD3- stimulated samples. The figure was made in dChip [21]. The color scale in the lower part of the picture corresponds to the mean expression of a gene. The red color represents expression level above mean expression of a gene across all samples, the white color is mean expression and the blue color represents expression lower than the mean.

The comparison of Itk-deficient and CsA-treated Wt T-cells revealed 482 up- or down-regulated transcripts upon anti-CD3-stimulation. In order to identify putative NFAT-binding sites (GGAAA), we selected 24 genes for bioinformatic analyses. The genes were chosen as being highly regulated in the CsA or Itk^-/- ^comparisons after anti-CD3-stimulation. 19/24 of these genes were also significantly regulated after co-stimulation with anti-CD3/CD28. 15/24 genes had putative NFAT-sites in the 500 bp region upstream of the transcriptional start site (Additional file 12). We identified 1 to 2 binding sites in 4 of those genes: *IL7R, Bub1, Ctla2a *and *Ctla2b*, as well as upstream of the translation initiation of *Schlafen1 (Slfn1) *gene (Figure 5a). To test whether NFAT binds to the promoter region of the genes *in vivo*, chromatin-immunoprecipitation experiments were performed. As a positive control, we used the IL-2 promoter region known to contain functional NFAT-sites bound by NFATc1 [34,35]. ChIP assays demonstrated NFATc1 binding to the IL-2 promoter region as expected and revealed anti-CD3 induced binding of NFATc1 to the selected regions of the five genes. They were also shown to be *bona fide *calcineurin-regulated genes owing to that the induced binding was reversed by CsA-treatment (Fig. 5b). A heat-map presenting the signal intensities of the above mentioned genes is shown in Figure 5c.

### Transcripts regulated by Tec-family kinases

Itk is crucial for T-cell development and activation. Similarly, Btk is essential for proper differentiation and activation of B-cells [36,37]. In a previous study, we investigated the genes modulated by Btk [24]. There was a pronounced overlap 18/38 (47%) between differentially expressed genes from the Itk-defective T-cells (900-list) and the previously published list of Btk-deficient genes from splenic B-cells as analyzed by the U74Av2 chip with approximately 12 000 genes [24]. The overlapping transcripts are shown in Table 7. Sixteen of the 18 genes were similarly regulated, which shows a highly significant co-variation (p = 0.01). Among these genes were those for transcription factors (Id2, Ikaros and Spi-C), cell membrane spanning (Csf1r, Mrc1 and Vcam1) and secreted proteins (Aif1, Igf1 and Tgfbi).

**Table 7:** Overlapping transcripts between Btk^-/- ^B-cells and Itk^-/- ^T-cells

**U74Av2**	**MOE 430 2.0**	**Gene Symbol, Gene Title**	**Fold change Btk^-/-^/Itk^-/-^**
102330_at	1418204_s_at	Aif1, allograft inflammatory factor 1	7.47/2.28
95546_g_at	1419519_at	Igf1, insulin-like growth factor 1	6.5/2.46
103226_at	1450430_at	Mrc1, mannose receptor, C type 1	5.42/6.06
95597_at	1423414_at	Ptgs1, prostaglandin-endoperoxide synthase 1	4.68/2.43 **
95597_at	1436448_a_at	Ptgs1, prostaglandin-endoperoxide synthase 1	4.68/2.19 **
96020_at	1417063_at	C1qb, complement component 1, q subcomponent, beta polypeptide	4.57/6.6 ***
96020_at	1434366_x_at	C1qb, complement component 1, q subcomponent, beta polypeptide	4.57/5.87 ***
96020_at	1437726_x_at	C1qb, complement component 1, q subcomponent, beta polypeptide	4.57/4.46 ***
104354_at	1419873_s_at	Csf1r, colony stimulating factor 1 receptor	4.4/3.96 **
104354_at	1423593_a_at	Csf1r, colony stimulating factor 1 receptor	4.4/2.95 **
103736_at	1448005_at	Sash1, SAM and SH3 domain containing 1	3.97/6.79
103454_at	1418555_x_at	Spic, Spi-C transcription factor (Spi-1/PU.1 related)	3.93/3.75 *
103454_at	1449134_s_at	Spic, Spi-C transcription factor (Spi-1/PU.1 related)	3.93/8.5 *
92877_at	1415871_at	Tgfbi, transforming growth factor, beta induced	3.9/3.46 ***
92877_at	1437463_x_at	Tgfbi, transforming growth factor, beta induced	3.9/5.04 ***
92877_at	1448123_s_at	Tgfbi, transforming growth factor, beta induced	3.9/4.52 ***
92877_at	1456250_x_at	Tgfbi, transforming growth factor, beta induced	3.9/4.6 ***
92223_at	1449401_at	C1qc, complement component 1, q subcomponent, C chain	3.73/5.47
92558_at	1448162_at	Vcam1, vascular cell adhesion molecule 1	3.63/5.62
102065_at	1418243_at	Fcna, ficolin A	3.6/5.27
103070_at	1416985_at	Sirpa, signal-regulatory protein alpha	3.42/3.58
102860_at	1424923_at	Serpina3g, serine (or cysteine) peptidase inhibitor, clade A, member 3G	3.05/2.08 ***
99476_at	1453931_at	Col14a1, collagen, type XIV, alpha 1	2.95/(-)2.51
93013_at	1422537_a_at	Id2, inhibitor of DNA binding 2	2.77/2.03 **
102293_at	1421303_at	Ikzf1, IKAROS family zinc finger 1	(-)2.7/(-)2.2
99413_at	1419610_at	Ccr1, chemokine (C-C motif) receptor 1	(-)4.13/2.62 *

The down-regulated transcripts are shown with "(-)"

* Denotes significant changes in Itk^-/- ^T-cells. * p ≤ 0.05 ** p ≤ 0.01 *** p ≤ 0.001

## Discussion

For any analysis of individuals with defective genes there are important considerations related to the choice of accurate controls and the adequate interpretation of the data. This is nicely exemplified in Itk-deficiency. Thus, when mice with Itk-deficiency are immunized and generate impaired responses it is unclear to what extent the impairment is caused by the reduced numbers of mature T-lymphocytes as compared to the increased innate populations versus that the mature as well as the innate populations are deficient because they lack Itk. The net outcome is the sum of these alterations. The same is true in microarray experiments or when phenotypic markers are assayed by other means. In the likely event that the innate populations themselves are further altered owing to lack of Itk, the corresponding population may not even exist in the Wt. The same principle is true for any mutant gene, and it is important to be aware of this fact when interpreting data, including expression profiling, related to such defects. In this report we describe the phenotypic changes in Itk-deficiency and make comparisons to CsA-treatment. Owing to the very large number of genes with altered expression, we here provide an overview of the observed changes. We pinpoint some of the interesting findings obtained from this dataset. However, the original gene profiling data, available to any investigators at GEO, could be analyzed in different ways, depending on the biological question to be answered.

T-cells deficient for the Tec-family kinase Itk have severe impairment during T-cell activation. Furthermore, Itk has also been shown to be involved in signaling pathways that regulate the development decisions of conventional versus innate-like T-cell development [9-13], since CD8^+ ^T-cells and a certain fraction of CD4^+ ^T-cells have an innate-like T-cell phenotype. Collectively, these studies revealed that Itk has a crucial and important function in T-cells. In this study we performed an Affymetrix microarray expression analysis to investigate how Itk-deficiency affects the expression profile in T-cells. The effect of Itk-deficiency was investigated in CD3^+ ^T-cells, as well as in the CD4^+ ^and CD8^+ ^T-cell subsets. These signatures for the first time reveal the transcriptome of Itk-deficiency.

The most pronounced changes were observed in resting Itk-deficient compared to Wt CD3^+ ^T-cells. This is in agreement with the previous findings that more genes are expressed in untreated cells as compared to stimulated T-lymphocytes [38-40]. Thus, after anti-CD3/CD28-stimulation the number of differentially expressed transcripts was dramatically decreased in Itk-defective (down by approximately 50%) compared to Wt cells. This suggests that the CD28 co-stimulatory pathway is less dependent of Itk. It was previously shown that Itk was a negative regulator of CD28-signaling in CD4^+ ^T-cells [41]. However, sorted naïve CD4^+ ^T-cells from Itk-deficient mice had normal CD28 co-stimulatory responses when compared to Wt cells [42], showing that CD28-signaling is not dependent on Itk in these cells. Our result confirms that Itk is not essential for CD28-signaling and suggests that a great deal of the TCR signaling defects in Itk^-/- ^T-cells is rescued by CD28 co-stimulation *in vitro*. However, expression of genes that is essential for T-cell proliferation like *Il2 *remain Itk-dependent after co-stimulation.

It was satisfying to observe the most pronounced transcriptional changes in CD8^+ ^cells, since Itk-deficiency is known to predominantly affect this subpopulation [9-11]. The overlap between CD4^+ ^and CD8^+ ^subsets was highest in untreated cells indicating an innate-like pattern also of the CD4^+ ^population. Moreover, a recent paper showed that CD4^+ ^T-cells in Itk-deficient mice have a memory phenotype with expression of typical surface markers such as CD62L^low ^and CD44^high ^[13]. When looking at the specific transcripts for each T-cell subset we found differences in expression of Klrs, two members in CD4^+ ^and four in CD8^+^. Klrb1a (Ly55a) and Klrb1c (NK-1.1) were found in CD4^+ ^T-cells. To our knowledge, only NK-1.1 was previously reported for Itk-deficient CD4^+ ^T-cells [13]. Klrc2 (NKG2C) and Klrk1 (NKG2D) were previously reported in CD8^+ ^T-cells [11]. In addition, we found Klra4 (Ly49D) and Klra19 (Ly49S), not previously described in this context. In unstimulated Itk^-/- ^CD3^+ ^T-cells eleven Klr members were found (shown in Table 3). Klra3, a7, a8 and b1b were shown to be common to the CD4^+ ^and CD8^+ ^subsets. Interestingly, we found Klra3 and Klra7 to also be calcineurin-dependent, while Klra8 was only Itk-dependent. Of note is also that a cytosolic protein known to characterize NK-cells, granzyme M, was present in the data. It has recently been shown to be expressed in NK-cells and cytotoxic T-cells with innate immune function [31]. Here, we show for the first time up-regulation of this transcript in CD8^+ ^Itk-defective T-cells. As expected, more differentially expressed genes were revealed following separation into the CD4^+ ^and CD8^+ ^subsets. In a mixed population changes that affect both subsets in a similar way are preferentially detected.

Itk-deficiency partially mimicked CsA-treatment, since there was a large overlap of affected transcripts. However, we observed that CsA had a much greater effect on transcriptional regulation compared to loss of Itk, especially after co-stimulation. Approximately 4000 genes were affected by CsA following either anti-CD3- or anti-CD3/CD28-stimulation, while the corresponding numbers for those also affected by Itk was 482 and 184, respectively. 113 probe-sets were shared between Itk-defective and CsA-treated T-cells independent of stimulation. Among them we found *Zbtb16*, encoding the transcriptional regulator PLZF, and *Xcl1*, which is also called lymphotactin or ATAC, a chemokine mainly produced by activated CD8^+ ^T-cells [43,44]. Also, *Crabp2 *was found in this comparison showing its calcineurin-dependency. *Crabp2 *is involved in regulating access of retinoic acid to its nuclear receptors, is developmentally regulated [45], and has been implicated in various forms of tumors. In addition, our analysis revealed that some of the Itk-induced changes are independent of the Ca^2+^/calcineurin pathway (322 and 225 transcripts after anti-CD3- and anti-CD3/CD28-stimulation, respectively). In this study we did not treat Itk-deficient cells with CsA. Such experiments could give further insights into the calcineurin-dependent regulation.

One interesting example of how different members of a gene family are differentially affected by Itk-deficiency and CsA-treatment is provided by the Granzyme family. Granzymes are serine proteases expressed in cytotoxic lymphocytes [46]. Interestingly, Granzymes A and K were both Itk- and calcineurin-dependent, while granzymes E and M were found to be Itk-dependent and calcineurin-independent after anti-CD3-stimulation. Both granzymes A and K induce caspase-independent cell death. Not much is known about granzyme E, while granzyme M is known to induce cell death in a caspase- and mitochondria-independent way [46]. Granzyme B was only affected in CsA-treated samples and has been shown to be involved in the induction of caspase-dependent apoptosis. Collectively, this demonstrates that expression of granzymes is differentially controlled.

The comparison of Itk-deficiency and CSA-treated CD3^+ ^T-cells led also to the identification of novel NFAT target genes. The combination of a bioinformatics approach and chromatin-immunoprecipitation assays revealed that *IL7R, Schlafen1, Bub1, Ctla2a and Ctla2b *are novel Itk- and calcineurin-dependent genes with seemingly functional NFAT-binding sites. However, they can be regulated in different ways, e.g. Ctla2a and Ctla2b, IL7R and Slfn1 were negatively regulated, while IL-2 and Bub1 were positively regulated by CN-dependent pathways. The same regulation pattern was observed in unstimulated Itk-deficient samples, but after anti-CD3-stimulation IL7R and Slfn1 became positively regulated by Itk (Fig. 5c). Members of the Schlafen (Slfn) protein family have been implicated in the regulation of cell growth and T-cell development. Furthermore, similar to the *Il2 *gene [47], AP-1 and NF-κB are reported to regulate Slfn2 expression [48]. Bub1 (budding uninhibited by benomyl) is a serine/threonine kinase that has a function in the mitotic spindle checkpoint and is mutated in certain types of human cancer [49]. Ctla2a and Ctla2b are cysteine proteinase inhibitors expressed in activated T-cells and mast cells [50]. Both naïve and memory T-cells have high levels of IL7R, and IL7 is required for their homeostasis [51]. Furthermore, recently it was shown that Wt and Itk-deficient CD4^+ ^T-cells express similar levels of IL7R (CD127) [13]. Certain genes in the Itk/CN group did not have *bona fide *NFAT-sites as determined by our bioinformatic approach. This could be due to that the current data base algorithms are not good enough to predict the putative sites or that the chromosomal stretches harboring NFAT-sites are located outside the 500 bp region that we choose to study. Future studies will aim to reveal a possible link between the altered expression of these genes and the phenotype of Itk-deficiency.

Finally, a comparative analysis of Itk-deficient T-cells and Btk-deficient B-cells revealed a significant overlap of transcripts, indicating that there is a common Tec family gene expression profile in lymphocytes. The fact that 16/18 genes had a similar fold-induction in T- and B-cells (p < 0.05) suggests common regulation of these genes by Itk and Btk. Conversely, the observation that two transcripts (Col14a1 and Ccr1) were differentially expressed may simply reflect that B- and T-cells represent different lineages, each characterized by unique features of their transcriptomes. Of the six most up-regulated genes in Btk-deficiency [24] all of them were >2-fold changed in cells lacking Itk, eight of which were also significantly altered in Itk-deficient T-cells (with p-values ranging from <0.05 to <3 × 10^-5^). Tgfbi, which was up-regulated in Btk^-/- ^and confirmed as highly increased in Itk^-/- ^(p < 3 × 10^-5^) samples, encodes an extracellular protein that mediates cell adhesion to collagen, laminin and fibronectin via its interaction with integrins [52]. Since these 16 genes are common to both Btk- and Itk-dependent transcriptomes it seems likely that the corresponding promoters could be activated through signaling components controlled by either pathway. Future identification of regulatory elements targeted by common factors could reveal the underlying mechanism.

## Conclusion

This report is the first to define the transcriptional signature of cells from Itk^-/- ^mice. The transcriptome of Itk-deficient cells revealed that there is a large overlap with regular CD4^+ ^and CD8^+ ^cells. Future studies analyzing different stages of innate, memory-like cells from Wt mice will aid in unraveling to what extent the innate population of Itk-deficiency also shows unique features, which differ from normal mice.

## Authors' contributions

Contribution: KEMB. designed and performed the majority of the research, analyzed the data and wrote the paper; NB. did animal experiments and cell stimulations; JML. designed and helped analyzing the microarray data, and helped with writing; LY. performed the ChIP experiments; JR. helped with the animal experiments and cell stimulations; AB. helped with the animal experiments, cell stimulations and writing; WE. was involved in the planning and execution of the project, and helped with writing; CIES. conceived project, provided supervision throughout, interpreted data, and helped with writing.

## Supplementary Material

Additional File 1 **Antibodies used in the isolation of CD3^+ ^T-cells**. Antibodies used in the isolation of CD3^+ ^T-cells. Click here for file

Additional File 2 **Primer sequences and PCR conditions used in the chromatin-immunoprecipitation assay**. PCR conditions and primer sequences for the genes *IL2, IL7R, Schlafen1, Bub1, Ctla2a and Ctla2b *used in the chromatin-immunoprecipitation experiment. F, forward primer; R, reverse primer and T (a), annealing temperature. Click here for file

Additional File 3 **106 immune response-related genes**. The list of 900 probe-sets that were differentially expressed between unstimulated Itk-defective and Wt T-cells was used to manually annotate 106 immune response-related genes. The genes were further divided into 14 different subgroups. The down-regulated transcripts are shown with "-". Click here for file

Additional File 4 **252 differentially expressed probe-sets in Itk-deficiency compared to Wt after anti-CD3- and anti-CD3/CD28-stimulations of CD3^+ ^T-cells**. All the comparisons were made against Wt and with the criterion ≥ 2-fold. Only 5/252 probe-sets showed opposite fold-changes in anti-CD3- and anti-CD3/CD28-stimulations for 24 h. These genes are marked with *. The down-regulated transcripts are shown with "-". Click here for file

Additional File 5 **3713 probe-sets overlapping upon anti-CD3- and anti-CD3/CD28-stimulations in Wt CD3^+ ^T-cells**. 3713 differentially expressed probe-sets overlapped in Wt CD3^+ ^T-cells after both anti-CD3- and anti-CD3/CD28-stimulations for 24 h. The comparisons were made against unstimulated Wt (Wt C) and with the criterion ≥ 2-fold. The paired Student t-test was used to calculate the p-values. The down-regulated transcripts are shown with "-". Click here for file

Additional File 6 **2393 probe-sets overlapping upon anti-CD3- and anti-CD3/CD28-stimulations in Itk-defective CD3^+ ^T-cells**. 2393 differentially expressed probe-sets overlapped in Itk^-/- ^CD3^+ ^T-cells after both anti-CD3- and anti-CD3/CD28-stimulations for 24 h. The comparisons were made against unstimulated Itk^-/- ^(Itk^-/- ^C) and with the criterion ≥ 2-fold. The paired Student t-test was used to calculate the p-values. The down-regulated transcripts are shown with "-". Click here for file

Additional File 7 **Eomesodermin and T-bet signal intensities**. Bar charts showing the mean signal intensity (MSI) levels of Eomesodermin and T-bet in CD4^+ ^and CD8^+ ^T-cell populations (**a and b**) and in CD3^+ ^T-cells (**c and d**) from Wt and Itk-deficient mice. Eomesodermin is represented by two probe-sets (grey and white bars). The two T-cell subsets are either unstimulated or anti-CD3-stimulated, while the CD3^+ ^T-cells are either unstimulated, anti-CD3- or anti-CD3/CD28-stimulated. Click here for file

Additional File 8 **324 differentially expressed probe-sets represent the core group of unstimulated Itk-defective CD4^+ ^T-cells**. The core group of probe-sets differentially expressed in unstimulated Itk^-/- ^CD4^+ ^T-cells (Itk^-/- ^CD4^+ ^C) compared to unstimulated Wt CD4^+ ^T-cells (Wt CD4^+ ^C). The comparisons were made with the criterion ≥ 2-fold. The unpaired Student t-test was used to calculate the p-values. The down-regulated transcripts are shown with "-". Click here for file

Additional File 9 **4467 differentially expressed probe-sets represent the core group of unstimulated Itk-defective CD8^+ ^T-cells**. The core group of probe-sets differentially expressed in unstimulated Itk^-/- ^CD8^+ ^T-cells (Itk^-/- ^CD8^+ ^C) compared to unstimulated Wt CD8^+ ^T-cells (Wt CD8^+ ^C). The comparisons were made with the criterion ≥ 2-fold. The down-regulated transcripts are shown with "-". Click here for file

Additional File 10 **113 probe-sets being both Itk- and calcineurin-dependent upon anti-CD3- and anti-CD3/CD28-stimulations in CD3^+ ^T-cells**. 113 probe-sets are Itk- and calcineurin-dependent upon anti-CD3- and anti-CD3/CD28-stimulations for 24 h. All the cells in the comparisons are CD3^+ ^T-cells. The comparisons were done using the criterion ≥ 2-fold and the p-values were calculated using unpaired (for Itk^-/- ^vs Wt) and paired (for Wt CsA vs Wt) Student t-tests. Cyclosporin A-treated samples are named CsA in the table. The down-regulated transcripts are shown with "-". Click here for file

Additional File 11 **95 probe-sets being Itk-dependent and calcineurin-independent after anti-CD3- and anti-CD3/CD28-stimulations in CD3^+ ^T-cells**. 95 probe-sets, corresponding to 89 transcripts, are Itk-dependent but calcineurin-independent upon anti-CD3- and anti-CD3/CD28-stimulations for 24 h. The comparisons were done using the criterion ≥ 2-fold and the p-values were calculated using unpaired Student t-test. The down-regulated transcripts are shown with "-". Click here for file

Additional File 12 **24 genes found in the bioinformatic searching for putative NFAT-sites**. The 24 genes were selected as highly regulated from the list comparing Itk^-/- ^anti-CD3- and Wt CsA anti-CD3-stimulated versus Wt anti-CD3-stimulated samples. 15/24 genes had NFAT-sites and six of them were verified to bind NFATc1. The genes that have NFAT-sites are grey-shaded and the genes verified to bind NFATc1 by chromatin-immunoprecipitation (ChIP) are in red. The genes marked with * are also found in the 113-list (Additional file 10) where genes are Itk- and calcineurin-dependent after both anti-CD3- and anti-CD3/CD28-stimulation. The down-regulated transcripts are shown with "-". Click here for file
