# Supplementary material for: Transcriptional signatures of Itk-deficient CD3+, CD4+ and CD8+ T-cells
Source: BMC Genomics. 2009 May 18;10:233. doi: 10.1186/1471-2164-10-233 (PMC2689280; doi:10.1186/1471-2164-10-233)
Supplement: Additional File 1 — Antibodies used in the isolation of CD3+ T-cells. Antibodies used in the isolation of CD3+ T-cells. [file 1471-2164-10-233-S1.doc]

**Additional file 1.**

| **Antibodies** | **Company** |
| --- | --- |
| bio-anti-CD11b (M1/70) | BD Pharmingen |
| bio-anti-CD11c (HL3) | BD Pharmingen |
| bio-anti-CD4 (CT-CD4) | Caltag |
| bio-anti-CD8α (5H10) | Caltag |
| bio-anti-CD45R (B220) | Caltag |
| bio-anti-Ly-6G (RB6-8C5) | Caltag |
| bio-anti-erythroid cells (Ter119) | Caltag |
| bio-anti-NK1.1 (PK136) | Caltag |
