# Supplementary material for: Transcriptional signatures of Itk-deficient CD3+, CD4+ and CD8+ T-cells
Source: BMC Genomics. 2009 May 18;10:233. doi: 10.1186/1471-2164-10-233 (PMC2689280; doi:10.1186/1471-2164-10-233)
Supplement: Additional File 2 — Primer sequences and PCR conditions used in the chromatin-immunoprecipitation assay. PCR conditions and primer sequences for the genes IL2, IL7R, Schlafen1, Bub1, Ctla2a and Ctla2b used in the chromatin-immunoprecipitation experiment. F, forward primer; R, reverse primer and T (a), annealing temperature. [file 1471-2164-10-233-S2.doc]

**Additional file 2.** Primer sequences and PCR conditions used in the chromatin-immunoprecipitation assay.

| Gene | F primer | R primer | T(a) | Cycles |
| --- | --- | --- | --- | --- |
| *Il2* | 5’-acctaagtgtgggctaaccc-3’ | 5’-gcatgctgtacatgcctgca-3’ | 55°C | 45 |
| *Il7R* | 5’-tgggacaactctatcctagc-3’ | 5’-cagaaacaggaagtctgagc-3’ | 56°C | 35 |
| *Schlafen1* | 5’-gtgaggatttcagctcggga-3’ | 5’-agagacatctggactctgtg-3’ | 56°C | 35 |
| *Bub1* | 5’-tcgtgagtgactctcaagac-3’ | 5’-aggtgctgactgagctaagg-3’ | 56°C | 40 |
| *Ctla2α* | 5’-ctgctgtgagacactgtagc-3’ | 5’-tggtacttgaggagagcagc-3’ | 56°C | 35 |
| *Ctla2β* | 5’-gagtcttggcattgtcagag-3’ | 5’-gaggctagtggtgcatgagg-3’ | 56°C | 40 |

PCR conditions:

95°C 5 min

94°C 30s

T(a) 30s 35-45 cycles

72°C 30s

72°C 10 min

4°C ∞
