# Supplementary figures and images for: Transcriptional signatures of Itk-deficient CD3+, CD4+ and CD8+ T-cells
Source: BMC Genomics. 2009 May 18;10:233. doi: 10.1186/1471-2164-10-233 (PMC2689280; doi:10.1186/1471-2164-10-233)

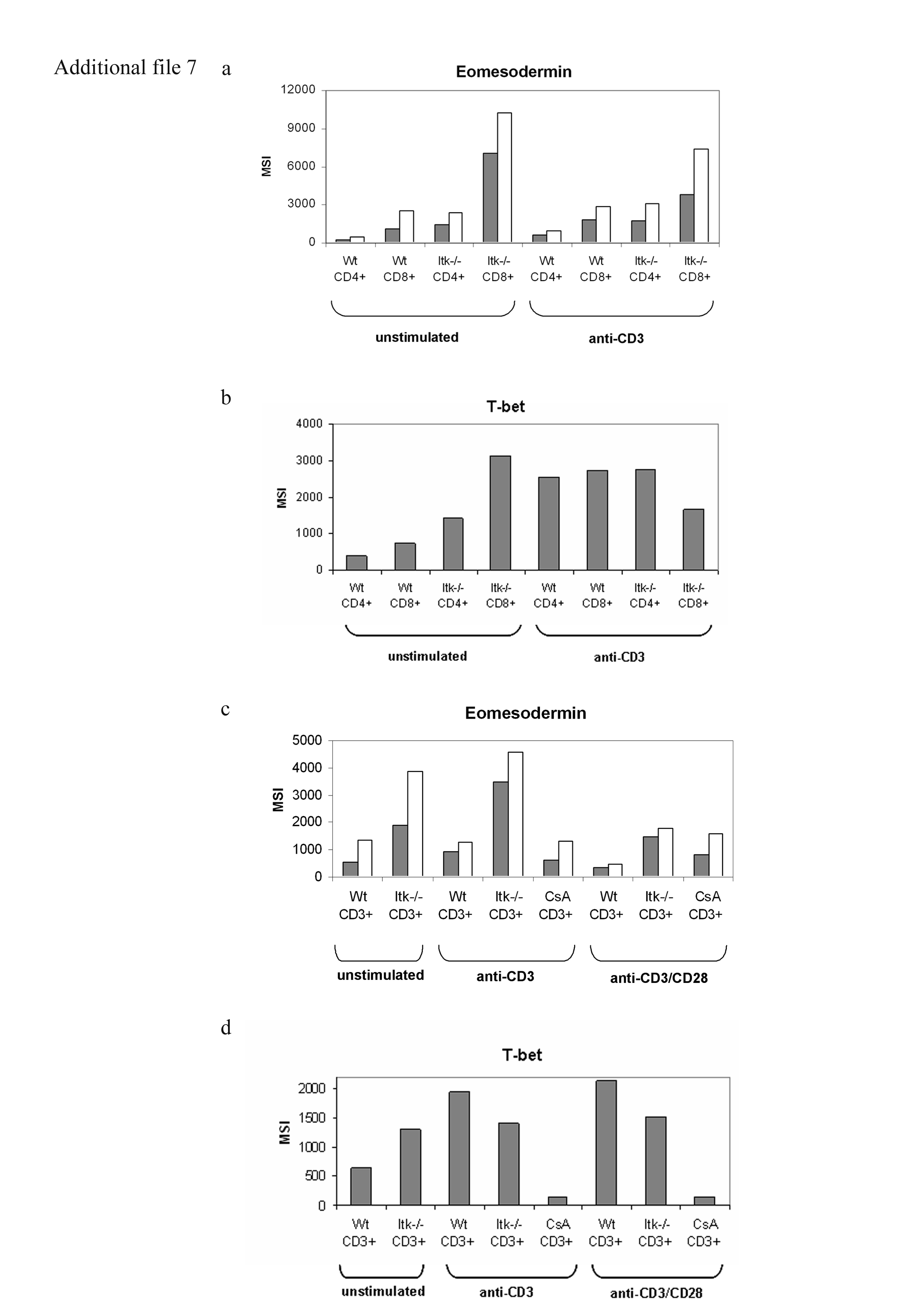

Supplement: Additional File 7 — Eomesodermin and T-bet signal intensities. Bar charts showing the mean signal intensity (MSI) levels of Eomesodermin and T-bet in CD4+ and CD8+ T-cell populations (a and b) and in CD3+ T-cells (c and d) from Wt and Itk-deficient mice. Eomesodermin is represented by two probe-sets (grey and white bars). The two T-cell subsets are either unstimulated or anti-CD3-stimulated, while the CD3+ T-cells are either unstimulated, anti-CD3- or anti-CD3/CD28-stimulated. [file 1471-2164-10-233-S7.tiff]
